# Patient involvement in rare diseases research: a scoping review of the literature and mixed method evaluation of Norwegian researchers’ experiences and perceptions

**DOI:** 10.1186/s13023-022-02357-y

**Published:** 2022-05-31

**Authors:** Gry Velvin, Thale Hartman, Trine Bathen

**Affiliations:** TRS National Resource Centre for Rare Disorders, Sunnaas Rehabilitation Hospital, 1450 Nesoddtangen, Nesodden, Oslo, Norway

**Keywords:** Patient involvement, Rare disease research, Methodology, Impacts, Benefits, Challenges, Effectiveness

## Abstract

**Background:**

Patients’ involvement (PI) in research is recognized as a valuable strategy for increasing the quality, developing more targeted research and to speed up more innovative research dissemination. Nevertheless, patient involvement in rare diseases research (PI-RDR) is scarce. The aims were: To study the Norwegian researchers` experiences and perceptions of PI-RDR and review the literature on PI-RDR.

**Methods:**

1. A systematic scoping review of the literature on PI-RDR. 2. A cross-sectional questionnaire study with close-ended and open-ended questions to investigate the researchers` experiences.

**Results:**

In the scoping review 608 articles read in full-text and 13 articles (one review and twelve primary studies) were included. The heterogeneity of the design, methodology and results was large. Most studies described several benefits of PI, but few described methods for measuring impacts and effectiveness of PI-RDR. In the cross sectional part of this study, 145 of 251 employees working in the nine Norwegian Centers on Rare Diseases participated, of these 69 were researchers. Most (95%) of the researchers claimed that rare diseases research is more challenging than for the more common diseases. The majority (95%) argued that PI-RDR may increase the quality of the studies and the relevance, and most (89%) agreed that PI-RDR in dissemination may increase the awareness and public interest for rare diseases. In the open-ended questions several researchers also claimed challenges related to PI-RDR, and many had proposal for improving PI and promotion of rare disease research.

**Conclusion:**

Both the literature and researchers emphasized that PI-RDR is important for improving research quality and increase the public attention on rare diseases, but what constitutes effective PI-RDR still remain unclear. More research on the design, methodology and assessment for measuring the impact of PI-RDR is warranted.

**Supplementary Information:**

The online version contains supplementary material available at 10.1186/s13023-022-02357-y.

## Background

Current estimates indicate that there are close to 7000 rare diseases (RDs) in the world [1, 2], and affect an estimated 30 million Europeans and 300 million people worldwide [1]. Most RDs have a genetic etiology, resulting in several chronic and progressive signs and symptoms [3]. Approximately 95% of RDs have no approved treatment [4], and RDs create significant challenges for affected individuals, their families, health and social care systems and society as whole [3, 5]. Despite that RDs have gathered more international attention the past decades, RDs are still much less studied than more common diseases [5]. RDs are associated with limited published research to inform interdisciplinary clinical practice, thus limited evidence-based practice, and barriers for developing clinical guidelines [3]. This scarcity of RDs knowledge is challenging for professionals who want to provide evidence-based health care to patients with RDS.

Modern clinical research increasingly recognize patient engagement (PI) as a valuable part of RDs research [3, 6]. Involving patients with RDs in the planning, conducting and dissemination of research may be a valuable approach for addressing evidence gaps for management of rare diseases. PI in research may promote research that evaluates health outcomes that are both relevant to patients with RDs and useful for decision making [3, 7–9]. Several Norwegian associations as well as most research funds in general have begun to require a statement of PI in their call for research proposals. According to INVOLVE [10] PI refers to the inclusion and activation of patients as partners in various stages of the research process, or as “research being carried out “with” or “by” patients rather than “about” or “for” them. Involving patients with rare diseases is emphasized as particularly important due to the low incidence and the unique challenges they face associated to the rarity of their diseases.

Studies [8, 11–13] indicate that Patient Involvement in Rare Diseases Research (PI-RDR) can improve the relevance of research questions, study design, methodology, recruitment rare, interpretation of data and financing. Thereby, higher likelihood of translation and adoption of research results in everyday practice as well as more effective communication findings. All of this may lead to improve clinical practice and better outcomes in patients [14]. Despite the growth of PI in rare diseases research and in general research, and that most researchers recognize the potential value of PI, achieving this potential in practice may involve many challenges. While literature is growing on different methods of PI, the conceptual meaning behind PI still in unclear. Lack of clarity of the definition and that the terminology of the concept varies greatly [15] may be a barrier to fully implement PI in rare diseases research. In a newly published review [15] of the definition of “patient involvement” or “patient engagement”, a horizon scan of related terms identified 24 terms each with multiple definitions across the health care sector. This review [15] found that one of the most common term used was patient involvement (PI).

Although there are anecdotal indication that PI-RDR increase the relevance and quality of studies, a challenge is the lack of systematic evidence to demonstrate the impact [8], and the limited amount of systematic sets of measurement methods for assessing the impact [14]. A systematic review from 2014 [8] of PI-RDR, including both articles and grey literature, found that most studies reported perceived impacts of PI-RDR that was not measured or confirmed. This review [8] also reported that studies mainly emphasized how PI could facilitate relevant clinical questions and patient centered outcomes, and few studies addressed PI-RDR in dissemination process. Improvement of communications around RDs may be essential to improve the quality of life for people with RDs [16]. The European Union Committee of Experts on Rare Diseases (EUCERD) recommendations for Centers` of Expertise (CoE) underscore the importance of collaboration with patient organizations in research and provide information that is accessible and adapted to the patients’ needs [17, 18].

Although authors report benefits of PI in the research process in general, the knowledge about approaches, impact and effectiveness of PI-RDR is limited. The existing PI framework have not addressed the specific considerations surrounding RDs [3]. Therefore, an overview of the existing research and more systematic information about the researchers` experiences and perceptions of PI-RDR seems warranted.

Therefore the aims of the study were:

1. To review and synthesize pertinent literature about Patient Involvement in Rare Diseases Research, identify research gaps, and discuss direction for further research.
2. To examine a group of Norwegian rare diseases researchers` perception and experiences with Patient Involvement in Rare Diseases Research.

## Methods and materials

### Aim 1: Review of literature on patient involvement in rare diseases research

#### Study design

A scoping review methodology was applied because this is a suitable method for mapping findings from a research area that is heterogeneous in methods or disciplines. Scoping reviews are also suitable for examining the extent, variety, range and characteristics on a topic such as Patient Involvement in Rare Diseases Research, and also for identifying gaps [19, 20]. The method is suitable for applying broad review questions, also when very diverse findings make systematic review with critical appraisal and meta-analysis difficult [20, 21]. This scoping review was performed according to recommendations from the Johanna Briggs Institute and Collaboration Centers guidance for conduction scoping reviews [22], and the PRISMA Extension for Scoping Reviews (PRISMA ScR) [21] (shown in Additional file 1). However, we have made one adjustment by looking into the findings of the included studies, and have synthesized the overall results on experiences, benefits and challenges of PI in rare disease research.

This review was guided by the question: “What is the characteristics and extent of research on Patient Involvement in Rare Diseases Research”.

1. What is the extent of secondary research articles (i.e. reviews) versus primary articles describing PI-RDR?
2. What is the characteristics of the study population (types of diseases, number of participants) and when and where have the studies been carried out (i.e. publication years and country)?
3. What types of PI approaches were used?
4. What was the reported impact of PI (on design, conduct, relevance, dissemination and impact on the participants) and effectiveness of PI (long-term outcome, cost-benefits etc.)?
5. How was the impact and/or effectiveness of PI measured (which methods or tools were used for assessments)?

#### *Eligibility* criteria

The review included secondary and primary research aiming to investigate and present results on Patient Involvement in Rare Diseases Research (adult patients, patient representatives and representatives for rare diseases organizations). Studies of PI on pediatric participants were not included due to the particularly methodology used for involving children in research. We included articles that stated that they aimed to investigate PI in Rare Diseases Research. Table 1 present inclusion and exclusion criteria.

**Table 1:** Inclusion and exclusion criteria

Inclusion criteria	Exclusion criteria
*Population*	
Adults (> 18 y) patients with rare genetic diseases, their representatives or parent to children with rare diseases, including as partner in rare disease research	People with other diseases than rare genetic diseases
Studies with participation from any country	Studies with broader populations not giving separate results of > 80% of adults with genetic diseases
	Studies mainly addressing paediatric patients, professional or other stakeholder as partner in rare diseases research
*Type of publications*	
Peer reviewed articles	Conference abstracts, commentaries, essays, consensus statements, book chapter reports, brochure, economic analyses,
Original research, primary studies	Articles dealing with legal or ethical issues, unpublished data (grey literature), study protocols, guidelines or non- systematic reviews
Secondary research studies: reviews	
All types of study designs	
*Topic of interest*	
Studies presenting results on patient involvement in rare diseases research	Articles dealing with legal or ethical issues, unpublished data (grey literature), study protocols, guidelines or non- systematic reviews
At least one aim Is to evaluate and describe patient involvement as a method in research	Studies addressing other issues than patient involvement in rare diseases research
Studies describing Patients involvement, but denotes the concept with another terms	
*Language*	
English, French, German, Danish, Norwegian, or Swedish language, including a English summary/abstract	Any other language

#### Search strategy

Systematic searches were conducted until 20th September 2021, in the following databases PubMed, AMED, CINAHL (EBSCO), Embrase (OVID), Eric, Google Scholar and Web of Science. We searches for a combination of subject heading (where applicable) and text words for rare diseases or rare disorders. We also searched for a combination of subject heading (where applicable) and text words for patient involvement and related terms with equivalent meaning (see Table 2). A three stage search strategy was utilized, including search words shown in Table 2. Search 1: rare diseases OR rare disorders. Search 2: patient involvement and words with equivalent meaning. Search 3: combining search 1 and search 2.

**Table 2:** Search strategy

1. Search with the following search terms: rare disease OR rare disorders 2. Search with the following search terms: Patient Involvement OR Participatory Research OR Integrated Knowledge Translation OR Community Participation OR Patient Engagement OR Patient and Public Involvement OR Co-production OR Knowledge Translation OR Consumer Involvement OR Patient Organizational Involvement OR Users Involvement OR Patient Partner research OR Patient and Service Engagement OR Collaborative research OR Patient Partner Research Or IKT OR End Of Grant IKT OR Knowledge Translation OR Knowledge To Action OR Knowledge mobilization OR Patient Partnership Research 3. Finally, we combined searches 1 and searches 2 Additional references were sought by examining the citations in included articles and consulting expert	

#### Selection of publications

All review steps were performed by two authors (TB/GV). Endnote software was used for selection and data extraction. Following the removal of duplicates, citations were screened independently by two reviewers (GV, TB) based on title and abstract (level 1 screening) and full-text articles (level 2 screening). The studies were assessed against the inclusion and exclusion criteria. When agreement was not reached, conflicts were resolved through discussion with a third author (TH), using the inclusion and exclusion criteria (Table 1). The screening process was done in two steps. In the first step abstracts and titles were reviewed and all articles clearly not meeting inclusion criteria were excluded. In second step full-text was collected and reviewed for the remaining articles, those that did not meet the eligibility criteria were excluded.

#### Data *extraction*

A data-extraction form was developed to provide a standardized and transparent method for mapping the methodology and findings from the studies [19, 20]. This was piloted on a subset of relevant papers and refined to ensure the extraction template met the specific objectives of the review. One researcher (GV) extracted data into a priori form, and another (TB) checked the accuracy. The following data were collected from each article. Bibliographic data, nationality/country of the study, study aim, participants data (number, diagnoses, age, status (patients, patient representatives, patient organization representatives or other stakeholders), objectives with PI-RDR, PI approaches and methods, reported impact of PI, measurement methods for measuring the impact (benefits and disadvantages) and effectiveness (long term utility, cost-benefits) of PI. The findings were summarized and synthesized [23] and presented in a table, and research dissemination was specified in a separate column due to the special methodology this entails.

### Aim 2: Norwegian rare disease researchers’ experiences and perceptions of patient involvement in rare diseases research

#### Organizational context and participants

All employees working in the nine Centers of Rare Diseases (CRDs) in Norway were invited to participate in this survey in November 2019. The nine CRDs are organized under the umbrella organization the National Advisory Unit on Rare Disorders in Norway (NKSD) [24]. NKSD is a governmental entity with a national responsibility to provide patients, medical health professionals and the general public with updated information on RDs. The CRDs play a central role in generating medical and psychosocial scientific knowledge on RDs Information about NKSD and the nine CRDs in Norway are available at website [24].

#### Study design and research questions

This cross sectional survey is a part of a larger study about patients’ involvement in research on rare diseases research, approved by the Data Protection Authority at Sunnaas Rehabilitation hospital (03.02.2019). The survey is conducted in accordance with the STROBE guidelines for observational studies [25]. A web-based survey was chosen to facilitate access and maintain anonymity of the participant.

The survey was guided by the question: “What was the researchers’ experiences and perceptions with Patient Involvement in Rare Diseases Research?”.

Our specific questions were:

1. Is there particular challenges related to rare diseases research?
2. What is the extent and experiences with PI-RDR?
3. What is the researchers` perceptions and experiences of benefits and challenges with PI-RDR.

#### Mixed method design

A study specific web-based questionnaire was developed due to lack of relevant validated instruments. The questionnaire had a mixed-method approach, combining closed-ended and open-ended questions strategically placed throughout the questionnaire. Mixed method is recommended as a powerful tool for collecting more detailed and specific responses from the respondents [26, 27]. The use of open-ended questions enabled us to give the respondents “another” response option, and to explore, explain or reconfirm the closed-ended questions. Analyses of the respondents’ verbatim responses gave important insight, not only into respondents’ substantive answers, but also in how they understood the questions and thereby establishing the validity of the questionnaire (26.27).

#### The questionnaire

The questionnaire was constructed in order to capture the employees and researchers experiences and perceptions of PI-RDR. The literature review was used to inform the choices of questions. The questionnaire was piloted by researchers in the Research Department at Sunnaas Rehabilitation Hospital, which led to valuable feedback and revision of three questions.

The questionnaire was divided into items of questions to all employees and specific questions to researchers only.

Questions to all employees:

- Questions on demographic data: Gender, age, educational level, profession, work place (rare disease center), length of employment history in the field of rare diseases.
- Two close-ended about their experiences with rare disease research.
- Two close-ended and three open-ended questions about the need for more knowledge about PI-RDR and proposal for improving PI-RDR and research dissemination.

Specific questions to researchers:

- Five close-ended and three open-ended questions to elicit the researchers’ perception and view of rare diseases research, and collect information of their suggestion for improving rare disease research.
- Three close-ended and three open-ended questions about experiences, perception and proposal for improving PI-RDR.
- Five questions including a 10 points Likert scale of the researchers experiences and perception of benefits and challenges related to PI-RDR and dissemination.
- Two close-ended and two open-ended questions about the researchers’ experiences and perception of benefits and challenges related to PI-RDR in dissemination and implementation of research results.

#### Analyses

A digital self-reported questionnaire Questback platform was established for data collection and analyzes [28], in addition to Excel. Questback software [28] is appropriate for capturing anonymized questionnaire responses digitally, facilitating automatic transcription and computer assisted coding. The quantitative data were analyzed and presented with descriptive statistics (mean, median and range or number and frequency, as appropriate). Thematic template analyses [29] were used for the open-ended questions, including a five steps process: (i) Get the data into the template by using excel, (ii) Identify response categories, (iii) Record the individual responses, (iv) Organizing the categories, (v) Present the data visually (using figures and quotations). Two researchers (GV/TH) independently conducted the initiating steps of the analytic process, of identifying categories and recording the individual responses into the categories. Discrepancy or disagreement were discussed until agreement with the third author (TB).

#### Ethical considerations

Ethical issues were considered in all stages of the study [30]. Invitation to participate was send to each of the employees` e-mail with information about the study and that participation was voluntarily, including a Web-link to the survey. The participants consented by voluntary returning completed questionnaire. Due to that the participants were employees from different CRDs in Norway and easily identifiable in the context, a detailed description of the sample and advanced statistical analyses were not conducted.

## Results

### Aim 1: Review of literature on patient involvement in rare disease research

**Fig. 1:**
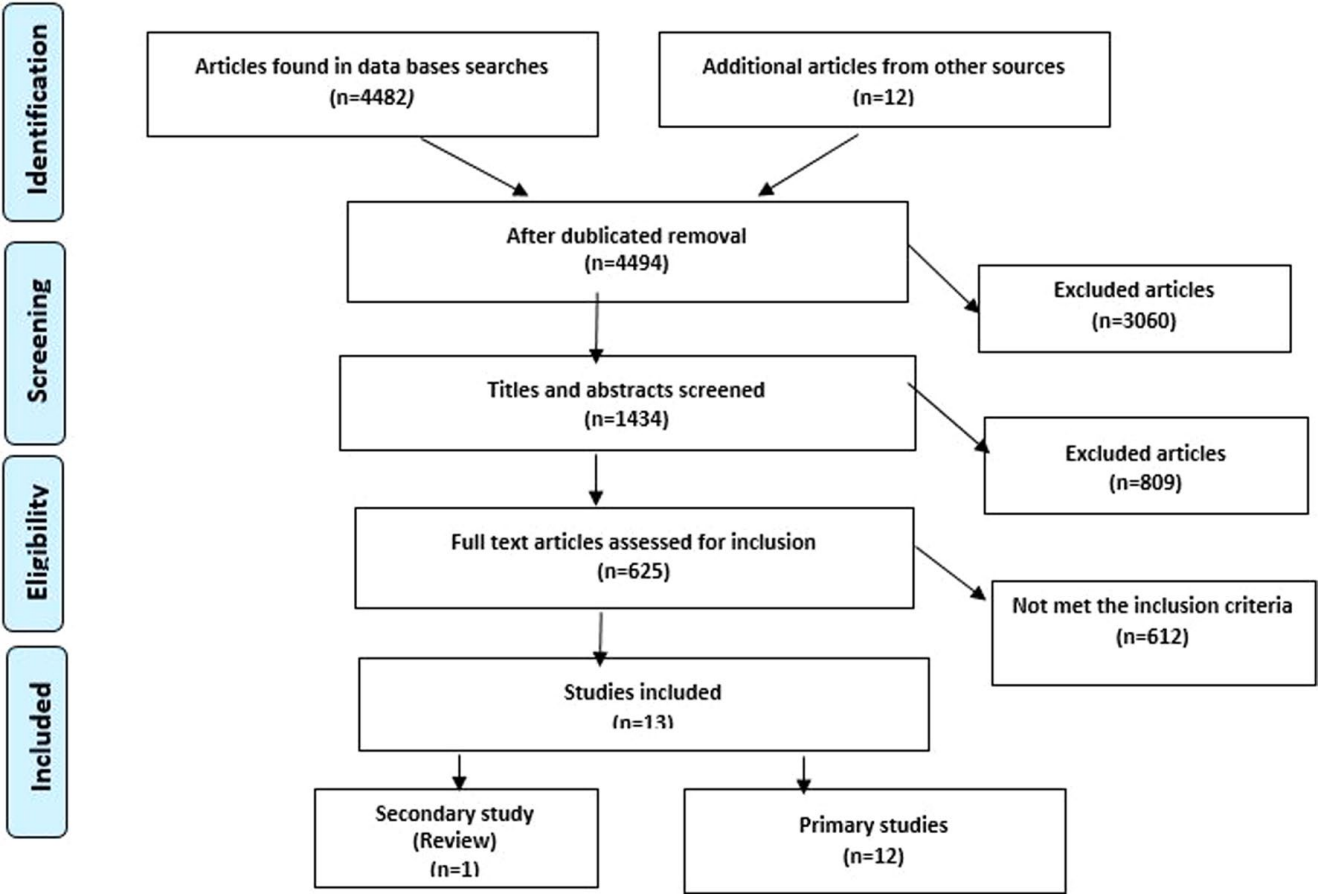
Flowchart of search, screening and inclusion process of scoping review

Of the 625 potentially relevant papers read in full text, 13 articles; one secondary (review) and twelve primary research articles met the eligibility criteria and were included in this review. Figure 1 shows a flowchart of the screening and inclusion process.

Data extraction from the thirteen included articles are shown in Table 3.

**Table 3:** Data extraction from included publications on patient involvement in research and research dissemination

*Publication details* First author Publication year Title Journal Country	*Publication information* Study design Aim of the study	*Patient involvement (PI) in the study* Patients’ condition(s) Number of rare disease patient representatives in the project Context/ organization of patient involvement	*Description of patient involvement in the rare disease research (PI-RDR)* Methods for PI in the study Methods for evaluation of the impact of PI Impact of PI Reported effectiveness/cost benefits of PI	*Description of patient involvement in rare disease research dissemination (PI-RDRD)* Information about PI-RDRD (yes/no) Methods for PI-RDRD? Methods for evaluation of PI-RDRD? Impact of PI-RDRD Effectiveness (cost-benefits) of PI-RDRD
*Review article*				
Forsythe et al. [8] A systematic review of approaches for engaging patients for research on rare diseases J Gen Intern Med USA	Systematic review, without quality assessment To summary evidence of involving patients and other stakeholders in research of rare diseases, included the role of the patients-organizations of rare disease have in promoting patient centered research	The review was guided by a multi-stakeholder technical expert panel (TEP) composed of patient representatives (no details on numbers and diagnoses), clinicians, and researchers recruited through the informal professional networks of the authors Review performed by the Patient-Centered outcomes research institute	Including different literature (n = 36) on PI in rare disease research, of these two [31, 37] fulfilled the inclusion criteria in this present study Patients were most commonly engaged in the preparatory stage for agenda setting (19 studies), study execution for study design and procedures (15 studies), for recruitment (12 studies), for data collection 6 studies. 19 studies described PI at multiple stages The review concludes that: No studies empirically evaluated the effect of patient engagement on the design, conduct, dissemination, or relevance of the research Many studies reported perceived impact of engagement that was not measured and could not be confirmed	Ten of the included studies reported PI in research dissemination The review concluded that there is limited information on how PI influence the dissemination and implementation of research results and influence the use of different dissemination channels Methods for assessing the impacts of PI were not described The study summarizing that several studies reported impacts of PI The review conclude that none of the included articles in the review had empirically evaluating the effect of PI in research dissemination
*Primary articles*				
Ambrosini et al. [32] “Be an ambassador for change that you would like to see”. A call to action to all stakeholders for co-creation in health care and medical research to improve quality of life of people with a neuro-muscular disease Orphanet Journal of Rare Diseases Italy and other European countries	Quality design with descriptive workshop report To report on a workshop to investigate the position of the neuromuscular patients community with respect to healthcare and medical research to identify and address gaps and bottlenecks	Patients and patients representatives with neuromuscular disorders, but also experts, industry and regularly bodies) 45 participants, but no detailed information on how many patients/ representatives Workshops—in Milano	*Shared decision making*- in discussions. *Partnership-based identification* -of wishes and needs of all stakeholders *The ladder of participation tool-* served as a model to evaluate the actual and desired level of patients’ involvement in all topics addressed.- the extent of the patients power to determining the end product- varying from a continuum of controlling- collaboration—consulting and—information) A consensus of the outcomes of the meetings was collected during the final plenary session Several examples were presented during the workshops showed that PI improve collaboration, compliance and commitment Did not describe the effectiveness (cost-benefits) of PI	No information on research dissemination in this articles
Badiu et al. [11] "Developing and evaluating rare disease educational materials co-created by expert clinicians and patients: the paradigm of congenital hypogonadotropic hypogonadism" Orphanet Journal or Rare diseases Switzerland, Belgium	Qualitative design with participatory research study To engage patients as partners to create high-quality Patent Education Material (PEM). Include evaluation the readability of PEM end end-user acceptability, as well as to disseminate these materials widely across different countries and cultures	Patient representatives with Congenital hypogonadotropic hypogonadism (CHH) /Kallmann syndrome No detailed information on how many patient representatives The PEM development was an iterative process involving multiple stakeholders including patients, patient support groups, clinicians and researchers spanning the fields of endocrinology, andrology, nursing and genetics	A community based participatory research framework was used to co-create PEM with patients; working in network groups for creating topics and consensus statement. Focus groups contributed to list of frequently asked questions, recurrent in social media and chat rooms, online evaluation of readability and acceptability, translation and cultural adapted to native speakers in different European countries Did not describe methods assessment of the impact of PI Describing that PI in co-creating patient-education material improved the understandability and actionability. PI enabled to create patient education materials that met patients-identified needs as evidenced by high end-user accessibility, understandability and actionability. Combining dissemination via traditional health care professional platforms as well as patient-centric sites can facilitate broad uptake to cultural adaptations Did not describe the effectiveness (cost-benefits)	Yes, describe PI In dissemination Expert clinicians, researchers and patients co-created the materials in a multi-step process. Six validated algorithms were used to assess reading level of the final product. Comprehensibility and actionability were measured using the Patient Education Materials Assessment Tool via web-based data collection No, the study do not describe methods for evaluating the impact of PI in research dissemination Describes the impact of PI on the use of different dissemination channels and that PI were important for translating into 20 languages. They concluded that **“**We believe that partnering with expert patients was an empowering experience and provides valuable contributions for developing patient-centered approaches to care” Did not describe effectiveness (cost-benefits) of PI
d’Ùdekem et al. [34] Involvement of patients and parents in research undertaken by the Australian and New Zealand Fontan Registry Cardiology in the Young Australia	Qualitative design descriptive reporting of experiences To describe the involvement of stakeholders in the development of the New Zealand Fontan registry	Patients and parents to children with Fontan circulation involved in the registry steering committee Number of patient representatives not described Registry created in at tight relationship with multiple stakeholders in the field of adult and child congenital heart disease in Australia and New Zealand	Have developed a model for PI, and describe different arenas PI research: Participation in the registry steering committee, creating of website and a Facebook page with updates and stories, meetings at Fontan education day, creating Advisory Committee-an advocacy group and web communication platform Did not describe methods for assessment the impacts Several benefits of PI: Safeguarding the project, control the messages given to families, adjust investigation and study feasibility, building a community, giving ideas for mew research, Increase participation rate, Increase research translation No description of the effectiveness I (cost-benefits)	Yes, describe PI in dissemination Describe how PI allowing early dissemination of research findings by multiple channels of communication. The focus was not only to provide information but also to give a voice to this community and include them as researchers. These communication channels are a part of a larger network involving the practitioner community, support groups, funding agencies, and health authorities. Describe PI in the use of different dissemination channels like social media, webpages, etc Did not describe methods for assessing the impacts of PI in research dissemination Described that PI creates more opportunities for research translation when it is closely connected to the patient community affected No description of the effectiveness (cost-benefits) of PI
Hamakawa et al. [13] The practice of active patient involvement in rare disease research using ICT: experiences and lessons from the RUDY JAPAN project Research Involvement and Engagement Japan and United Kingdom	Mixed method, quantitative questionnaire and web meeting To investigate the practice of active involvement of patients in medical research in Japan by utilizing a digital platform, and to analyze the outcomes to clarify what specific approaches could be put into practice	Patients with Skeletal muscle channelopathies and hereditary angioedema The number of participants not described A rare disease research platform that utilizes Information and Communication Technology (ICT) and Steering Committee meetings	Several approaches for PI were designed through patient-researcher collaboration, namely the Steering Committee, questionnaire development, dynamic consent, and other communication strategies. Patients were involved in: conceptualizing, governance, software system, questionnaire, recruitment, data use, analyzing and interpretation, communication and dissemination They described that they analyzed the research process of practices and experiences on how each approach of PI affected and contributed to the research project They reported increased self-efficacy for the patients involved and believe patients expertise influence research. They claimed that the research outcomes potentially provided better treatment/healthcare	Communication including dissemination of the progress and results of the research Practical methods for communication, using social media to increase the study external profile, help engage patients, building Web-sites and developing a Patient Network Forum Did no described methods for assessing impact PI reported to increase the use of a variety of communication strategies, including the Patient Network Forum, the website and newsletters Did not describe the effectiveness (cost-benefits)
Landy et al. [31] How disease advocacy organizations participate in clinical research: a survey of genetic organizations Genetic in Medicine USA	Quantitative cross-sectional study, with study specific questionnaire To examine how patients and patient organizations are involved in research of how the deliver	Not specified diagnoses other than that they were genetic or chromosomal in origin Leaders in 104 of 206 (62%) patients’ organizations of rare genetic conditions answered the questionnaire Invited through the Genetic Alliance`s Disease InforSearch	Description of PI in study design (56%), in recruiting (91%), data collection (75%), in preparing a research report (44%), and research analyses (37%) A questionnaire for reporting the experiences and feeling of the impact of PI-RDR(D) Leaders felt they had substantial and positive effect of PI, 65% felt that they had increased the amount of research relevant for their condition, 58% felt that PI had increased participation rates “a lot”, 48% reported PI-RDR as extremely important activities 68% felt their involvement in clinical research had increased the amount of research on their condition	Most Patient-Organizations reported that they had an active role in dissemination of research Patients were involved in different dissemination activities: 89% by web site or newsletter, 60% supported or organized scientific conferences, 31% reported helping disseminating through the press, and 30% had presented at scientific conferences Did not describe methods for assessment of impacts Did no describe any impacts Did not describe the effectiveness (cost-benefits) of PI
Lochmüller et al. [33] The position of neuromuscular patients in shared decision making Report from the 235th ENMC workshop: Milan, Italy, January 19–20, 2018 J Neuromuscul Dis Italy, and other European countries	Qualitative design with descriptive workshop report To report on a workshop to investigate the position of the neuromuscular patient community with respect to research settings: Level of PI in medical research for neuromuscular diseases: (i) registries and biobanks; (ii) clinical trials; and (iii) regulatory processes	Patient representatives with neuromuscular disorders 35 participants, but no detailed information on how many or which diseases Workshops including 35 participants mainly representing the patient community (also experts, industry and regularly bodies)	Works-shops with “*Partnership-based identification”* -of wishes and needs of all stakeholders. Patients were involved discussion of issues related to quality of life using the Shared decision making model, involved in discussion of registry and biobanks, clinical trials design. regularity and developing of Informed consents *The ladder of participation tool-* served as a model to evaluate the actual and desired level of patients` involvement in all topics addressed. A consensus of the outcomes of the meetings was collected during the final plenary session. Discussion and reflection between researchers and patients about benefits and the Description of method for measuring PI as evaluation and discussion in the process Several examples were presented during the workshops showed that PI improve collaboration, compliance and commitment. PI in fundamental to address what really matters, and creating awareness and engagement Did no describe effectiveness of PI, but described needs for improvements in order to facilitate higher patient involvement in research. Including three types of improvements (i) Educational changes; (ii) Cultural changes, and (iii) Structural changes	Only minimal description of PI in research dissemination
Merkel et al. [38] The partnership of patient advocacy groups and clinical investigators in the rare diseases clinical research network Orphanet Journal of Rare Diseases USA, international Network groups	Quantitative cross-sectional study with online questionnaire To outline the roles patients and patient advocacy groups (PAGs) play in the rare diseases clinical research network (RDCRN) and reports on the PAGs impact on the Network`s success	No detailed information on diagnoses Representatives from 28 of 76 patient advocacy groups (PAGs) associated to RDCRN participated., and researchers from 17 Research consortium in RDCRN participated Principal Investigators (PIs) from the 17 RDCRN Consortia and 28 representatives from 76 PAGs affiliated with these Consortia were contacted by email to provide feedback via an online RDCRN survey	Different approaches, 82% provided patients with education 89% PAGs reporting participating in protocol review, study design, consortium, conferences, attending consortium meeting. Responding PAGs reported PAG participation in protocol review, study design, Consortium conference calls, attending Consortium meetings, or helping with patient recruitment Did report using two survey of the researchers and the PAGs perception of the impact of PI The most frequently cited benefits of PIs were help with patient recruitment for RDCRN studies (73%), communication of Consortium activities within the patient community (40%), and providing direct funding to the Consortium (27%), participation in Consortium conference calls (54%), inclusion in Consortium activities (50%), and help with patient recruitment for RDCRN studies (46%) Did no describe effectiveness of PI (cost–benefit) of PI	PI in dissemination were described 96% were involved in disseminate information about Consortium activities within the patient community via their PAG websites, newsletters, and other forms of communication. 86% of PAGs include updates for their associated Consortium during their PAG- meetings. Most PAGs (82%) also provide patients with educational materials related to Consortium activities Methods for measuring the levels of involvement were described PI entailed more use of different dissemination channels such as social media, newsletters and other mean Did not describe the effectiveness (cost-benefits) of PI
Nunn et al. [35] Involving people affected by a rare condition in shaping future genomic research Research Involvement and Engagement Australia and New Zealand	Mixed method design with participatory action research To report the process, experiences and outcomes of involving people in genomic research in a standardized way, in order to inform future methods of involvement in research co-production	Patient representatives from an Australian patient organization for Eosinophilic gastrointestinal disorders was involved in co-design of the study 26 participated: 15 participated in the online discussion and 12 completed the follow-up survey > 18 years) Recruitment: Online community hosted by an Australian based rare disease charity	Patients were part of the study team and gave feedback on the proposed study design, involved in a number of tasks including reviewing and improving the written information, online survey questions, and the facilitation plan for the online discussion Describes methods for evaluating PI in research: ‘Standardized Data on Initiatives (STARDIT)—can be used to plan, report and evaluate involvement in research, including impact. An online survey before joining a two-week facilitated online discussion, followed by a second online survey Pi was reported to change the study design, including improving language used in recruitment and learning resources The effectiveness (cost-benefits) not reported	Very little information about PI in research dissemination Patients are involved in dissemination Did not describe methods for assessing impact Reported improved written information and more use of alternative channels for dissemination Assumed that PI made educational materials more useful and that patients wanted to be involved in further parts of the research process, like co-authoring scientific papers
Pinto et al. [39] The involvement of patient organizations in rare disease research; a mixed method study in Australia Orphanet Journal of Rare Diseases Australia	Mixed method study: analyses of the Patient organization web-sites, online web-based questionnaire and qualitative individual interviews To describe the characteristics of Australian RDPOs; evaluate their research ambitions and modes of involvement in research; and discuss the challenges that Australian RDPO leaders identify with their efforts to contribute to research	Diagnoses not described Leaders of 61 (of 114) Australian rare disease patient organizations answered questionnaire and 10 participated in interviews Centre for health Equity School of Population and Global Health, University of Melbourne, Australia were responsible for the study. They reviewed 112 RDPO websites, conducted an online survey completed by 61 organizational leaders, and interviewed ten leaders and two key informants	92% of the leaders reported that supporting research or promoting research was an important goal for the organization. 95% had also had undertaken research activity such as: financing, lobby A questionnaire survey was used for evaluating the impact of PI PI may improve lack of financial resources, lack of interest on rare diseases, increase the recruitment rate and registry. Therefore important that researchers must work with Patient-Organizations The study reported that PI is important and could help advance scientific knowledge and therapy, thereby alleviating the personal and societal burden of RDs	Described PI in research dissemination 78% reported being involved in disseminated of research, such as doing it available at the web site RDPO websites showed that leaders had compiled information about PI in current research projects and findings PI had increased the use of more relevant research dissemination channels Interviewed leaders gave examples of how this information had led organizational members to develop ideas for further research, which they then used in discussions with researchers or as a basis for awarding research funding
Roennow et al. [36]. Collaboration between patient organisations and a clinical research sponsor in a rare disease condition: learnings from a community advisory board and best practice for future collaborations BMJ Open International collaboration	Qualitative design with communication of experiences To share our experiences from such a collaboration undertaken surrounding the SENSCIS® clinical trial (NCT02597933), and discuss its impact during, and legacy beyond, the trial	Diagnosis: scleroderma / systemic sclerosis 15 patient representatives from Scleroderma patient associations from 10 countries Describes establishment of a community advisory board (CAB): a transparent, multiyear collaboration between the scleroderma patient community and a clinical research sponsor	A community advisory board was established where patient offer their expertise to clinical research sponsor to discuss overall program development. -The board reviewed and provided advice on trial conduct and reporting. outcomes of the collaboration in three areas: the implementation and conduct of the clinical trial; analysis and dissemination of the results; and aspects of the collaboration not related to the trial Did not described the methods for assessment of the impacts of PI They reports that PI led to improvement and optimization of trial procedures; meaningful, patient-focused adaptations were made to address challenges relevant to scleroderma-associated interstitial lung disease patients Did no described efficacy (cost-benefits) of PI	Written lay summaries were developed by the trial sponsor with valuable input from the CAB to ensure that language and figures were understandable and accessible to lay audiences and patients The CAB and the CRS also collaborated to co-develop opening tools for medication blister packs and bottles No description of assessment for measuring impact, but presented shared learning and discussions PI was reported important for raising awareness among physicians’, patients and caregivers, educational materials to improve diagnosis and management of scleroderma were co-created and delivered by the CAB and CRS Did not describe effectiveness (cost-benefits) of PI
Swartz et al. [37] Pachyonychia Congenita Project. A partnership of Patient and Medical Professionals Journal of Dermatology Nurses Association USA	Qualitative approach by involving patients in collaborating groups, to transform the researchers and clinicians understanding of PC and in symptoms A PC-project was set out to eliminate the barriers of isolation by creating an international collaborative network of patients, medical professionals and scientist to be a catalyst to find effective treatments for the disease	Patients with Pachyonychia Congenita (PC) The number of participants not described PC- project Using Web site, web-meetings, registry and databases, PC project has connected researchers’ interest in PC	Description of how to help patients find out what work for them. Patients report into a PC Wiki section available on the Web site, and tthrough Web site both patients and medical professionals can stay informed about opportunities to participate in educational meeting, Webinars and clinical trials. Patients contributed with personal stories, provided physician consultation, genetic testing through the International PC Research Registry Did not describe the methods for measuring the impact, but the professionals described how and what they had learned by PI Through this collaboration, researchers have made several discoveries that have transformed the understanding of the condition and treatment. Description on how the project launched its first annual PC Awareness Day in 2012 to begin connecting and empowering the PC patient community in its support of public awareness Did not describe the effectiveness, but could help better outcome for patients	Did describe PI in dissemination Describe PI in the use of different channels for dissemination such as educational meeting, webinars, developing a Web-site with the latest news, extensive images, a complete bibliography of scientific articles, patient education brochures, annual report,, including a Facebook page No description of assessment for measuring impact of PI PI entailed more comprehensive us of different communication channels PI in dissemination could increase the empathy and understanding of rare diseases in the general public, and thereby develop a more supportive environment for those experience rare diseases
Young et al. [12] Patient involvement in medical research: what patients and physicians learn from each other Orphanet Journal of Rare Diseases USA	Qualitative study with observational and qualitative individual telephone interviews of patients participated in VPPRN governance since 2014, and observational study This project examined the recently formed Vasculitis Patient-Powered Research Network (VPPRN), a rare disease research network, to better understand what investigators and patients learned from working on research teams together	Diseases with vasculitis disease 13 patient-partners with vasculitis disease participated, in addition 5 study mangers/staff, 4 MD or PhD investigators, Research observational notes from 6 in-persons and 42 telephone /web conferences meetings This qualitative study examined the results of 18 months of data collection for a project, funded by the PCORI	The patients were involved in all stages of the research projects description of how patient-partners and investigators characterized their working relationships with one another, what they learned from their collaborations, and provided recommendations for future teams of patient-partners and investigators The study used comprehensive methods for measuring and analyzing PI, using Interviews, in-person-meeting, observations, notes and telephone interviews They claimed that direct engagement in research design and development by patient-partners can result in a positive and productive working relationship for all members of a medical research team. This bi-directional engagement directly benefits and impacts research design, participant recruitment to studies, and study subject retention. Major themes included the great benefits of communicating about activities, being open to listening to each group member, and the importance of setting reasonable expectations Did not describe the effectiveness (cost-benefits) of PI, but claimed that network of patients can influence future research projects, review plans, ideas, and protocols for research studies, and to generate ideas for future research endeavors	Minimal description of PI in research dissemination The teams examined had goal of creating a network of patients to participate in further research studies Did not describe assessment for measuring impact of PI Did no reported impacts Did not describe effectiveness (cost-benefits), but claimed that PI can improve the effectiveness of dissemination

#### Secondary studies (review)

Only one review [8] of PI in research of rare diseases was identified, published in 2014. This was a systematic review, but no quality assessment of the included articles was conducted. This review included all types of publications (n = 35) (narrative reports, web-sites, qualitative studies, survey studies and grey literature), on patients, parents, professional and other stakeholders involvement. The authors of the review claimed that although nearly all included articles reported benefits of PI, the methods for assessment of the impact and effectiveness of PI were lacking. Only two [31, 37] of the 35 publications in this review fulfilled our inclusion criteria. The reason for the exclusion was that most of these publications were not peer reviewed articles and/or not dealt with rare diseases.

#### Primary studies

All 12 primary articles were all published in English, conducted between 2012 [31] and 2021 [35], and originating from USA (n = 4), Europe (n = 4), Oceania (n = 3) and Japan (articles = 1). The studies described PI in research of different types of RDs: neuromuscular diseases [32, 33], congenital hypogonadotropic Hypogonadism /Kallmann syndrome [11], Fontan disease [34], eosinophilic gastrointestinal disorder [35], scleroderma disease [36], vasculitis disease [12], Pachyonychia Congenita [37], skeletal muscle channelopathies and hereditary angioedema [13], and rare disease in general [31, 38, 39]. Both patients, patient representatives and patient organization representatives were involved. The number of PI participations varied from 13 [12] to 104 [31]. Four [11, 13, 34, 37] did not described the number of participants involved in PI. Few studies reported the age, gender or ethnicity of patient-representatives (Table 3).

The main objectives with most of the included articles were to create high quality research by using PI, to examine different methods of PI, and/or to share their experiences of the process, methods and perceived impact of PI and to address gaps.

#### What types of patient involvement approaches were used?

There was vide variation of design and approaches of PI in the different studies. Most articles [11, 12, 32–34, 36, 37] used qualitative design and three [13, 35, 39] had mixed-methods design, combining participatory research, surveys and online meetings. The PI-approaches were based on participatory active research methods, using work-shops groups, individual interviews, Delphi methods, participation in Consortiums, “partnership identification methods” and “shared decision making”. Two [31, 38] had quantitative cross-sectional design, using quantitative questionnaire and pilot-testing. Nearly all studies described PI in at least one stage of the research process such as including patients in the steering comities or Consortium [13, 34], creating research topic and design [11–13, 31–33, 35, 36, 38, 39], questionnaire development and consent [11–13, 35, 36], recruitment [12, 13, 35], data collection [11, 12, 31], analyzing and interpreting [12, 13, 31, 36], communication and dissemination [11–13, 31, 34–38], translation and cultural adaptation [11], financing and lobby [38, 39].

#### Reported impact of patient involvement in rare diseases research

All studies reported some positive impact of PI-RDR either on the process or the products, or both. Several studies emphasized the importance of including patients in the early stage and setting the research agenda, influencing new avenues for research, improving the study design and the research protocol and by addressing what really matters for patients [12, 33–35, 38, 39]. It was reported that PI-RDR is of great importance for improving the relevance and the utility of the study [11, 12, 33–35, 38, 39]. Three studies [12, 33, 38] also reported benefits of PI on recruitment rate by promoting research projects through patient organizations and web-sites. PI-RDR was also reported improving the compliance and commitment for both researchers and patients involved in the research project [32, 38]. Four studies [12, 32, 37, 38] also emphasized the benefits of mutually learning through interaction and new understanding and new perspectives as benefits of PI-RDR. One study [37] reported that researchers and clinicians made several discoveries that had transformed their understanding of the diseases and treatment, significant for diagnosing and counselling patients. Increased self-efficacy and confidence for the patients involved were also reported [12, 13, 37, 39]. Three studies [11, 34, 38] claimed that PI-RDR in the dissemination of research increased the end-use accessibility, understandability, readability and action ability. They indicated that PI-RDR in the translation and dissemination meant more effectivity in conveying the message and reaching the target audience. One study [33] reported that PI-RDR created more awareness and engagement in society and increased financial support. Another study [39] indicated that PI-RDR contributed to valuable insight into the difficulties or small organizations trying to do research.

Few studies described challenges and disadvantages with PI-RDR, but some [12, 13] described that PI requires extra time and efforts for patients and researchers. Descriptions of the effectiveness such as long-time utility or cost-benefits of PI were almost absent.

#### How was the impact and effectiveness of patient involvement in rare diseases research measured?

The studies stated different methods for measuring the impact of PI, but the measurement and methodology were very limited described in most studies [13, 31, 37–39]. Some [11, 34, 36] did not include any information about the methodology used for measuring the impact of PI-RDR. The majority of the studies mainly offered and account of experiences with PI in research, elaborated and presented jointly be academic researchers and patients, claiming that empowerment was an important purpose in each instance. One study [35] used a questionnaire; the Standard Data on Initiatives (STADIT) to researchers and patients about their experiences and perceptions of the process and impacts. Another [32] used the “Ladder of participation tool” for assessing the levels of involvement. Only one study [12] included comprehensive description of methods for assessing the process and impacts of PI-RDR, combining interviews, observation noted, web-conferences and telephone interview. Very few studies described in detailed what worked and which strategies generated desired outcomes, or rigorously assessed the research activities.

### Aim 2: Results from the mixed-method study of the researchers’ experiences and perceptions

Of 251 employees from nine Norwegian Center for Rare Diseases, 145 participated (response rate 58%). Of these; 77% were women, 54% > 50 years old, 62% had higher education (master or Ph.D), and 47% had worked more than 11 years with RDs. Forty-eight percent (N = 69) had conducted research on RDs (see Table 4).

**Table 4:** Participant demographics

N = 145	
Gender	
Woman	77%
Age	
20 to 39 years	16%
40–49 years	32%
50–59 years	26%
> 60 years	26%
Education	
< Bachelor	38%
Master degree	41%
Doctor degree	21%
Number of years in rare disease services	
0–5 years	39%
6–10 years	14%
> 11 years	47%
Have conducted research projects	
Yes	48% (n = 69)
No	52% (n = 76)

The results are presented sequentially by the themes from the questionnaire, including the categories that emerged from the open-ended questions for each of the themes. The four themes were: (1) Challenges with rare diseases research, (2) The extent of experiences with PI-RDR and perceived benefits and challenges. The themes including the subcategories, are illustrated by researchers’ citations (shown in Additional file 2).

#### Challenge in rare disease research

**Fig. 2:**
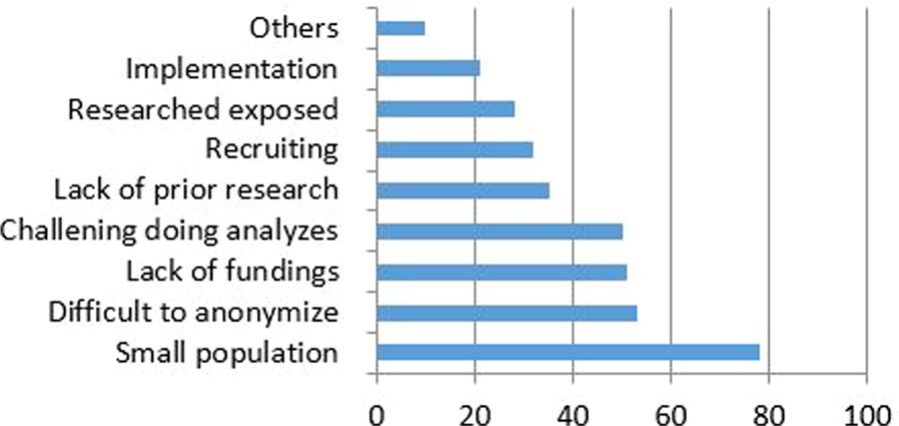
Challenges in rare disease research

Of the researchers, 95% (n = 65) reported more challenges conducting rare disease research, than research on more common diseases, such as: small population (79%), study design (56%), anonymizing of participants (55%), lack of funding (51%), statistical analyzes (50%) the lack of prior research (48%), and recruiting respondents (34%) (Fig. 2).

In the open-ended questions researchers reported that rare diseases research poses unique challenges, and that many barriers exists in advancing knowledge and recruitment options for RDs. The most obvious challenge mentioned for conducting rigorous research was the small sample sizes, but also the heterogeneity between and within RDs.

Some challenges are unique to rare diseases research, one is the small populations, another the heterogeneity, including the different subtypes of the diagnoses (researcher).

Some researchers also claimed that methodological and data constraints may limit the ability to generate evidence. Others emphasized the analytic challenges related to small samples, whether there is sufficient (statistical) power to draw definitive conclusion, included the extent to which available data can be viewed as representative for the entire population of patients within the condition.

We need particular design for rare diseases. It is difficult to do statistical analyses and there is a need for specific methods for rare diseases research (researcher).

Many of the researchers had proposals for improving rare diseases research. Some mentioned that it is important to identify innovative approaches that may overcome the methodological challenges inherent in the study of rare diseases. Other proposed international research collaboration on specific disease populations and particularly on the ultra-rare diseases, to increase the study population. Others stated a need for more collaboration within the rare society for developing more rigorous methodology, and illuminate the particular challenges related to living with rare diseases.

Together we are stronger- we need to work together, to find common solutions in the rare society (researcher)

#### The researchers’ experiences and perceptions of patient involvement in rare diseases research

Fifty-one percent of the researchers reported that they had conducted PI-RDR, and 54% stated that PI is of particular importance in rare diseases research. Their understanding of the concept of PI-RDR and dissemination varied, 32% defined PI as “that patients are informed about the project”, 30% that “the patients are involved and can give feedback”, and the rest that “the patients are actively involved in all phases of this research process”.

**Fig. 3:**
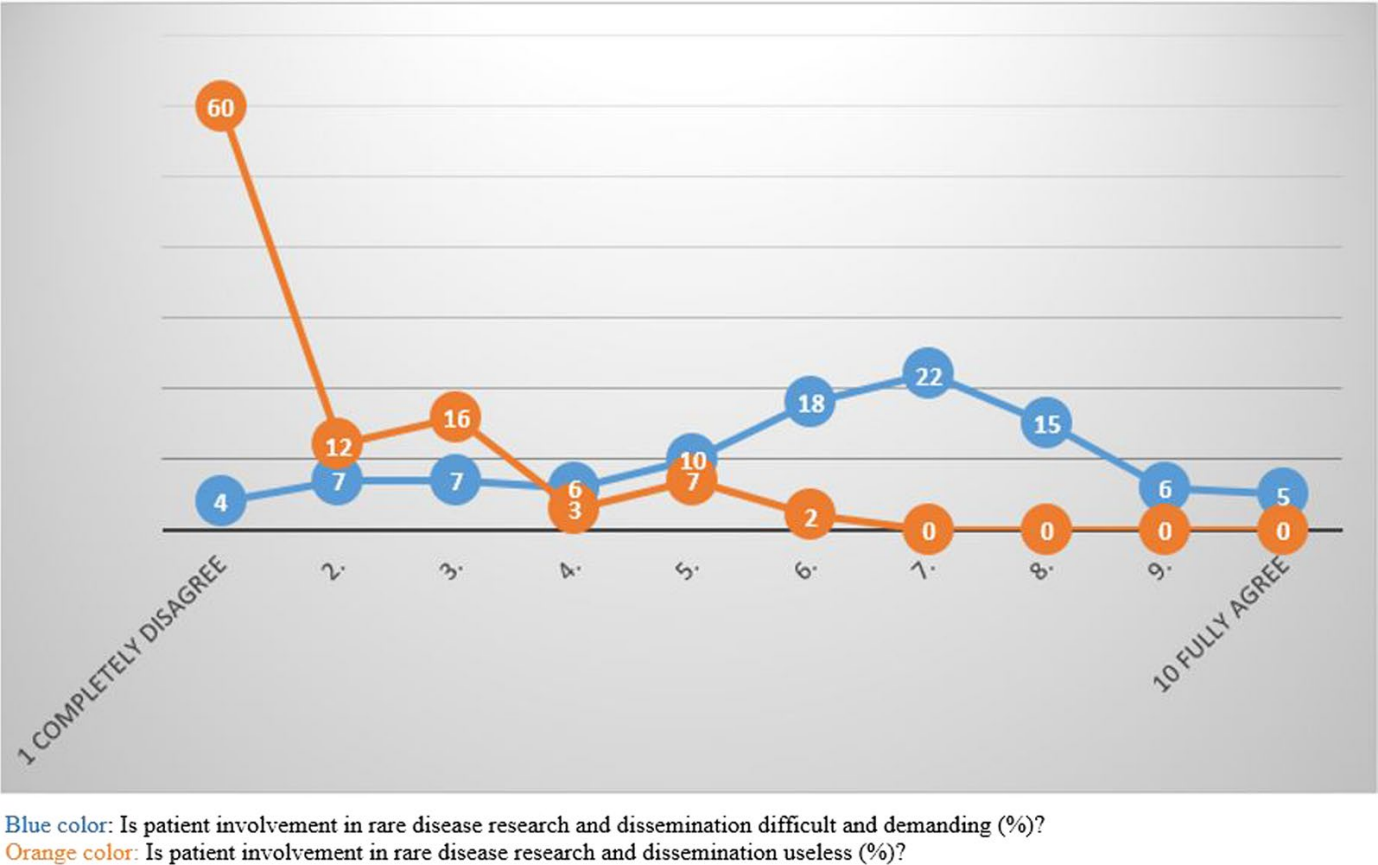
Is PI in rare disease research useless and demanding? (%)

On a-scale from 1 to 10 very few agreed that PI-RDR was useless, but several stated that it is difficult and demanding (Fig. 3).

In the open-ended questions most researchers were positive to PI-RDR. Several emphasized that is was important to involve patients in the early stage of the research process for identifying research topic that are relevant and address patients’ needs.

Collaboration with patients is crucial for high quality research. They know what they need so we have to listen (researcher).

Most of the researchers recommended PI throughout the research circle. Several mentioned the particular benefits of PI on the recruitment rate, due to small populations. They claimed that involving patients’ organizations may increase the legitimacy and interest of the study when patients’ organization promote information about research projects on their web-sites or meetings. Others stated that involving patients in the analyses and interpretation of results could provide new dimensions and understanding of the results. Some claimed that PI could help promote collaboration networks and provide financial support for research infrastructure. Others stated that it is important to plan levels and types of PI, appropriate for the different projects. Some stated that in some research projects PI may not be of great value, and that it is important to avoid PI as tokenism or just being “politically correct. The PI must be fruitful and meaningful for both the patients and the researchers.

Despite that PI is politically correct, the collaboration must be realistic compared to what we achieve. In some type of research projects PI may be meaningless.

Several researchers’ also stated challenges related to PI, such as difficulties in educating patients for equal research participation, power imbalance between researcher and patients and the importance of facilitating and paying attention to the patients’ health problems when they are involved in research process. Some also claimed that PI is resource-intensive, time-consuming and logistically demanding, therefore it is a necessary to assess the effectiveness and long-term utility impact of PI-RDR.

**Fig. 4:**
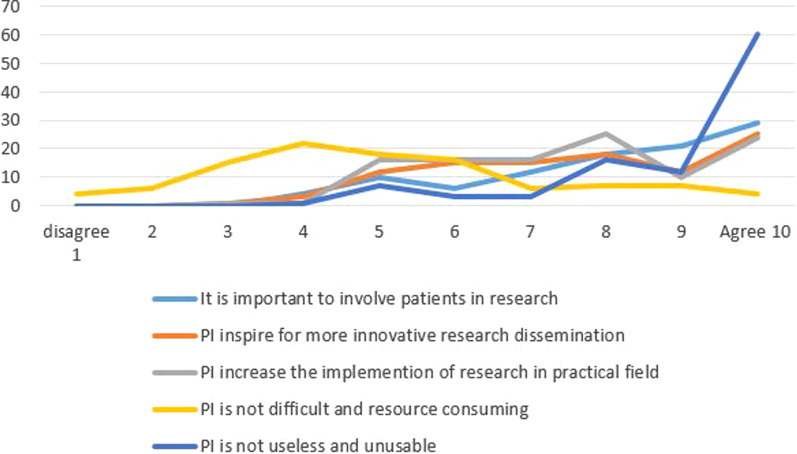
The utility of PI in research dissemination

Forty-one of the researchers reported that they had conducted PI-RDR in the dissemination process. Most, 89% agreed that PI can be a useful tool for increasing more relevant and targeted knowledge translation, and increase the use of more creative and innovative communications channels. Most (79%) claimed that the research dissemination in RDs is more difficult than for the more common diseases, due to lack of researchers and societal interest (see Fig. 4).

In the open-ended questions several researchers claimed that patients can assist in translating and communicating the research in a more accessible language, thus reaching out to a wider community more efficiently. Several stated that more appropriate communication can increase the understanding and awareness of the patients` needs, which in turn can lead to more relevant research projects on rare diseases.

When involving the patients, the research have more impact, as the researcher is grounded in an understanding and prioritization of patients need (researchers)

## Discussion

### Aim 1: The review of pertinent literature

#### The characteristics of the studies

Despite that PI-RDR is recognized as a valuable methods for increasing the quality and relevance of research, the studies on PI-RDR is very limited. Only 13 articles were included in this review, indicating that RDs still is much less studied than common diseases [3]. Most of the included studies were from the Western Countries, and more than half have been published the last three years. The cultural differences in involving patients in the research process may be of interest for further studies. All included articles emphasized the importance of examining and sharing their experiences of PI-RDR, its impact and address gaps. Most studies reported several positive benefits of PI-RDR, but the heterogeneity of PI-methods and methods for assessment of the impact of PI were large, and in most articles poorly described.

#### The reported impact of PI

In all the included articles both researchers and patients had cited positive impacts of PI; on the research itself, with the design, executing and dissemination as more applicable to those populations the research were intended to serve. Commonly reported impact of PI were more relevant research questions, improved design, methodology and increased recruitment rates, similar to reported in studies of more common diseases [40–42]. Some also described the empowerment aspects of PI-RDR, such as transformational learning outcomes both for researchers and patients, aligning with studies of more common diseases [43]. Indicating that transformational learning may occur through the process of involving patients in research.

Some also emphasized that PI-RDR may be a method to better deal with the particular challenges related to rare diseases research such as lack of interest, financing, awareness and policies support. When the number and size of research studies are small, as in RDs research, targeted outcomes and dissemination seems of particular importance [44–48]. To overcome this barriers Gagnon et al. [3] have introduced the Knowledge Mining methodology Framework (RKMMF) to improve the development of knowledge translation products and dissemination in RDs for developing clinical practice guidelines and evidence based practice. This methodological framework emphasize the patients` experiences as an important source of information, and can be a rigorous and flexible framework that can be adapted to the specific context of many rare diseases [3]. The authors [3] suggest that involving patients and end-users as early as possible may improve the dissemination products and meet the end-users need and expectations. This in accordance with some of the included studies that indicate that PI-RDR in dissemination may increase the acceptability, utility and relevance of the study results, and may increase the public awareness and policy support of RDs.

#### Methods for assessing the impact of patient involvement

Even though the international rare disease research network emphasize the importance of involving patients in research, [12, 34, 49, 50], our results indicate that the scientific proof of the impact and efficiency of involving patients in research is limited. The included studies did not go into detail in analyzing the nature of the process of PI that leads to impact. Few studies described strategies that generated desired or reported outcome. The transferability, strength of impact and relevance of the findings were often not extensively discussed. Preconceptions and founding values frequently seems to be confirmed, reappearing as results without substantial discussion and methodological reflection. Only one study [35] used a validated tool for measuring the process and impact of PI, most studies used reflection and discussion methodology and included minimal description of on how the assessments were conducted. The quality of the involvement process is a key [14, 51]. There is only with rigorous evidence about the impact of PI-RDR the researchers can makes strides towards exploring the best recipes for PI-RDR. Appropriate methodology for impact assessment seems to be lacking [14, 43, 51]. Therefore, more validated instruments and methods for assessing the process and impact of PI seems warranted. More systematic assessment of the different conceptual frameworks of PI-RDR could provide opportunities to evaluate and compare across a range of study design.

### Aim 2: The researchers’ perceptions and experiences with patient involvement

#### Experiences and challenges with rare diseases research

The results from our cross-sectional mixed methods study showed that nearly all researchers reported that research on RDs is more challenging than for the more common diseases. This align with other studies [3, 8, 37, 46], indicating that research on RDs poses unique challenges related to choice of study design, analyses, recruitment and assessment of the representativeness for the entire population. In RDs research, the most obvious challenge to conduct rigorous research is the small number of eligible participants [52, 53]. Other challenges reported were the heterogeneity between and within rare diseases, the geographical dispersion of patients, lack of knowledge about clinical course and lack of appropriate treatments [52, 53]. Several of the researchers had proposals for improving the study quality by increasing the patient population by international collaboration. The European Union (EU) has put much effort into funding rare diseases research, encouraging national funding organizations to collaborate together in the E-Rare program, setting up European References Networks for rare diseases, and initiating the International Rare Diseases Research Consortium (IRDiRC) together with the National Institutes of health in the USA [54]. The international collaboration in RDs may be the key to improve the life of people with RDs [54].

#### Experiences and perception of patient involvement in rare diseases research

Many of the researchers had experiences with PI-RDR, and nearly all emphasized the particular importance of PI in rare diseases research. They reported benefits such as: more relevant research questions and meaningful research, which may lead to improved study design, more effective methods, increased recruitment, higher quality of data, improved interpretation and higher likelihood of translation and implementation of the research into everyday clinical practice. This is similar to reported in the articles included in the review part of this article. Nevertheless, the researchers emphasized that there are also several challenges related to PI-RDR. Some reported difficulties in educating patients for equal research participation, this may create power imbalance between researchers and patients. Power imbalance or lack of real involvement may result in tokenism, indicating only making perfunctory or symbolic effort to involve the patients [55]. Some of the researchers also specified that PI is resource-intensive, time-consuming and logistically demanding, therefore the needs to assess the effectiveness and long-term utility impact of PI-RDR also was emphasized.

When asking the researchers to define PI, their perception of the concept greatly varied. Only one third of the researchers perceived PI as actively involving patient in all phases of the research and dissemination process, similar to the definition of INVOLVE [10]. This may indicate that PI is a complex phenomenon lacking a clear theory and definition, and impose problems because many fail to define PI. In a study of Boaz et al. [56] researchers were asked about their attitude towards PI. This study found that attitudes ranged from positive expectation that PI improves research, to PI being something that needs to be done to comply with a formal demand. Even if the patients are willing to get involved, the effective realization of PI can be hampered by different ideas of PI, or misinterpretation of aims and expectations among and between patients and researchers [56]. A mismatch can inhibit successful involvement. Therefore a common understanding of the concept of PI-RDR is important. Another aspect emphasized by the researchers in our study was that PI is not suitable for all types of studies, and it is important that PI-RDR is meaningful both for researches and the patients. Therefore planning PI in an early stage of the research process and finding appropriate PI-RDR methods were emphasized of several researchers in our study.

## Implication for further research

Despite that both the literature and the researchers claim several benefits of PI-RDR, our study indicate that there are some challenges related to PI that need further research. One challenge is that the concept of PI-RDR is complex and lacking a clear definition and theory. This lack of a clear definition may impose a problem, because many studies fail to define PI. Another challenge is the heterogeneity of PI methodology. PI is not a simple activity and it takes many forms and operate on many levels, and are highly contextual. It is very hard to standardize a PI-RDR method, and it is not a single simple intervention testable. Nevertheless, many studies describe that they have involved patients in all stages of the research circle, and experienced great benefits of PI-RDR. It had been advantageous if the methodology and approaches in these studies had been described in more detail. A third challenge is that validated measurements methods for assessing the impact of PI seems to be lacking. Most studies included in our review described positive benefits of PI-RDR, but very few described how they had measured these impacts. This may indicate that existing measures and approaches to evaluate the impacts of PI on the conduct of research and particularly on research outcome are limited. It is only by careful measurement of the impact of PI in research we truly can identify whether it ultimately benefits rare disease patients and improve the health care. Therefore more research on approaches and methodology of PI and PI-measurement in research is warranted, and particularly on PI in rare diseases research.

## Limitations and strengths

Our scoping review inclusion criteria, restricted to English, French, German and Nordic languages may have lost important references, however the broad search in several databases may have compensated somewhat for this. In addition, PI is not a well-defined concept and we may have missed some studies using other terminology than included in our extensive search terms. The PRISMA-ScR was followed, but the included articles were not assessed regarding risk of bias, and thereby the results should be interpreted with caution.

The cross-sectional survey of the researchers` experiences may have several potential biases. We were unable to do analysis on responders versus non-responders, due to lack of information in ensuring the anonymity of the participants. There may also be a possibility that we have not reached all employees working in the nine Center of Rare Diseases, however, the response rate was high. Study-specific questionnaires may imply possibilities for misunderstanding the questions. Nevertheless, the construction of a questionnaire was deemed necessary, as no validated questionnaire existed for the purpose of this study. The pilot testing of the questionnaire in the Research Department of Sunnaas Rehabilitation hospital has probably decreased potential biases. Retrospective questions might also have introduced recall bias to the results. The use of Questback online survey program made it possible to feed the responses into the data analysis software automatically, and this avoids associated data transcription error. A strength was the combination of close-ended and open-ended questions, but the mixed method analyses and presentation of result are complex and require thoroughly methodological knowledge, something we believe the authors possess.

## Conclusion

This study revealed that the research on PI in rare disease research is limited, but both the studies researchers seems to emphasize that PI is particularly important in rare diseases research due to the unique challenges related to research on small heterogeneous populations. The results from the included articles in the review and researchers experiences poses many positive impact of PI such as improving the quality of the research, and improved clinical relevance, designs, acceptability, legitimation and dissemination. However, appropriate methodology for PI seems not clear, and needs further definition and research.

Despite this challenges, the positive attitudes and experiences of patients, researcher and society indicate that PI in rare diseases has great positive impact. We therefore believe the benefits of this way of doing research outweigh the challenges.

## Supplementary Information

**Additional file 1.** PRISMA-ScR checklist.

**Additional file 2.** Summarizing of answers of the open-ended questions from the researchers in main themes and categories

## Data Availability

The dataset supporting the scoping review part of the article is included within the article (and its additional files), the dataset from the cross-sectional part is available in reasonable request to the corresponding author.
