# Supplementary material for: Patient involvement in rare diseases research: a scoping review of the literature and mixed method evaluation of Norwegian researchers’ experiences and perceptions
Source: Orphanet J Rare Dis. 2022 May 31;17:212. doi: 10.1186/s13023-022-02357-y (PMC9158134; doi:10.1186/s13023-022-02357-y)
Supplement: Supplementary file 2 — Additional file 2. Summarizing of answers of the open-ended questions from the researchers in main themes and categories [file 13023_2022_2357_MOESM2_ESM.docx]

| **Supplementary table 2**  **Summarizing of the open-ended questions from the researchers in the main themes and categories.** | | |
| --- | --- | --- |
| **Themes** | **Categories** | **Quotations from researchers** |
| **1. Challenges in rare research** | **Small patient population is a problem** | To achieve larger N  *-Some challenges is unique to rare disease research, one is the small population, and another is the heterogeneity, including different subtypes of the diagnoses.*  *-I think, small samples is the characteristics of the rarity, and thereby a huge problem in research.*  *-In small countries like Norway, the N is very small, may we should have more collaboration with the Nordic countries.*  *- It is necessary to collaborate internationally due to small populations.*  *-We need to think broader and global.*  *-It is difficult to cooperate across national borders, but we must strive to achieve this, especially in countries with few inhabitants.*  *-Ultra rare research depends on international collaboration.*  *-To increase then N, we need international collaboration.*  To emphasize the rarity of the disease  *-To find what is common and find common solution in the rare society.*  *-National collaboration between the nine centers are necessary.*  *-The rarity is the difficulty, that`s what we need to emphasize.*  *-Together we are stronger, and many people with rare diseases have same experiences.*  *-The rarity is something particular, that our common interest.*  *-We need a carrot to achieve more and better research dissemination.*  *-We need to work together, to find common solution in the rare society.*  *-National collaboration between the nine centers are necessary.*  *-The rarity is the difficulty, that`s what we need to emphasize.*  *-Together we are stronger, and many people with rare diseases have same experiences.*  *-The rarity is something particular, that our common interest.* |
|  | **Methodological challenges rare disease research** | *-Rare disease research poses challenges to investigate – it require specific approaches.*  *-We need particular design for rare diseases, it is difficult to do statistical analysis and there is a need for specific methods for rare research.*  *-There are several methodological challenges in rare disease research, and lack of statistical power require specific approaches and rigorous statistical-valid analyses.*  *-Limited amount of data available affect the feasibility of developing models that meet good practice.*  *-Doing research on rare diseases require particular research approaches, that need to be developed.*  *- A problem is analytic challenges- to which the available data can be viewed as representative for the whole population and whether it is sufficient statistically power to draw clinical decisions.*  -*There is a lack of disease-specific measurement developed for different rare diseases- more research on this is necessary.*  *-We can`t use ordinary HRQoL measurement, these instrument do not suitable for rare diseases, so it is a need for more consensus conferences and specialist cooperation.*  *-It is recognized that in small sample sized, the p-values are especially vulnerable to small deviation in the outcome.*  *-Together, we may develop suitable measurement for different diagnoses.* |
|  | **Lack of funding and prestige- of rare disease research** | -*The research on larger groups is given priority, We have to be creative, because it is difficult to get funding in rare research, think new and alternative!*  *-International research groups may increase the chance for funding.*  -*Many researcher do not prioritize to do research on rare disease, there is much more prestige in the more common conditions, so we have to encourage rare disease research.*  *-Despite that each condition is rare, all together rare diseases are common in the society, and thereby profitable investment for society. This we have to promote.*  *-Recruitment of high quality researchers are difficult in rare diseases, we might have to find ways of promote the research as important.*  *-Involving patients ensure the research accountability for the public.* |
| 2.**Patient involvement in rare disease research** | **To encourage for fruitful, substantial and enduring partnership between patients and researchers.** | *-Collaboration with the patients is crucial for high quality research. They know what they need.*  *-First of all, do good research. Patients and organizations are good communicators for research on their own diagnosis.*  *-Create joint projects that deal with several rare diagnosis, on special topics. Show that there are relatively many in Norway who have a rare diagnosis*  *-Despite that PI are political correct, the collaboration must be realistic compared to what we achieve. In some type of research it might not be appropriate to involve the patients.*  *-I think it is a bit problematic that the patients guiding and deciding the purpose and dissemination of research. They have a useful role in identifying research areas and areas that should be explored, and may make important contributions and input, but research should be independent.*  *-Exchange experiences and share success factors. Look for ways to tell user stories, case, without coming into conflict with what we are allowed to do*  *-Because health and social services have little interest in research of rare diseases, it is very importance that the research are relevant and useful for the practical field*  *-The patients know what they need and whom need the knowledge.*  *- PI may promote results that evaluate health outcome that are both relevant to patients and useful for decision makers*  *-By promoting the knowledge as relevant for more common diseases, more people will be interested. .*  *-PI is important, it is not about but with the patients.*  *-It is of crucial importance, and I have worked with several projects involving patients in research.*  *-Patients are excellent advocates for generating public interest.* |
| **3. Patient involvement in research dissemination of rare diseases** | **More impactful research with more targeted messages** | *-The patients can assist the creating of communication, translation, dissemination into accessible language to reach a wider community more efficiently.*  *-To greater extent collaborate with those who will use the knowledge - make the knowledge available in channels where those who need it find it. Make the knowledge accessible and easy to understand, and relevant to the user group – The patients are often the basis communicators of knowledge to the professionals.*  *-Appropriate communication will help with better understanding of patient needs.*  *-When involving the patients, the research become more impactful, as the research is grounded in an understanding and prioritization of patients’ needs.*  *-Collaboration with those who will use the knowledge can help sharpen the message and help us spread it out.*  *-Close collaboration with the relevant patient groups is the best channel for disseminating the knowledge. Particular the most active knowledge. Patients often reach out to another audience than the researchers.*  *-Participatory research can make the research results more updated and pertinent for the professionals and stakeholders.*  *-Due to the little extent of knowledge, it is important to involve the user in the whole research and the implementation process so that the knowledge are more useful and pertinent.*  ***-****They know how to do it, in a way that makes the research more relevant for the clinicians.*  *-The patients may be important for implementation of the research in practice.*  *-I think it is important to involve the users; the research dissemination may be more nuanced and balanced. The users see other aspects than the researchers in the dissemination.*  *-I think that dissemination of research results is very important and an area where user participation is not only necessary, but also meaningful. They have their angle on what is important to convey.*  *-User participation in research dissemination should be completely natural.*  *-It is an important topic. Users must participate in research dissemination because they can contribute information that makes the dissemination more targeted and relevant.*  *-I have a lot of thoughts about this. If the research cannot be communicated to users, the research is of less relevance and poorer quality.*  *-Hand-on information may contribute to more targeted message to the target groups.*  *-User-participation in research dissemination are new and exciting. I think that there are a lot to win on think how one could reach out with the results in a more popular scientific ways.*  *-Users may know alternative and better communication channels to reach out with rare research*  *-Users with rare diseases are often experts on their own diagnosis, and they know who need more knowledge and in which way.*  *-I think the User-Organizations is important in dissemination of the research through their web-sites, social media and their organizational paper.*  *-Important with popular science research dissemination with user participation, in parallel with scientific dissemination.*  *-The knowledge-users can find the right communication channels tailored to the right audience.*  *-Important to reach out, so that the research on rare diseases not are drowning in the research on larger groups.*  *- The might help us to think about alternative, to get out of our comfort zone, and try something new*  *In dialog we might find better more creative and better ways for reaching out with our important results.* |
|  | **Practical and ethical implications** | -*It is not easy, it might be a way of reaching out with the research, but it is important to take care of the patients in these processes.*  *-It is important that the patients get paid for the work they do, not only as unpaid volunteers ?*  *-The professional patients are often involved in several project, and may lack time and capacity to be involved enough to have real influence. We have to involve new ones.*  *-A communication plan should be prepared already when the project is being planned – influenced by the users.*  *-It is important to divide between scientific referee articles where the users should not influence and more popular scientific articles were the users are of great importance*  *The users need education and must have good insight in the issues of relevance*  *-User-participation is important but sometimes the requirements become too static, formal and difficult to meet.*  *-It requires a lot of training and resources from the users themselves. - Other audiences and professionals are research recipients, who use research as a tool to do their job.*  *-User participation in research dissemination is not incorporated as a routine with us and will require some work to be implemented*  *-There may be challenges around recruiting representative user representatives when you have few people with a diagnosis****.***  ***-****When we apply for REK, we should think about the research dissemination and possibly the use of project participants. It is conceivable that this must be applied for separately in relation to identification / anonymity etc*  *-It is important to respect that the users and the researchers have to complete different roles, competence, capacity, and time to participate, and it is important to respect that each of the parts brings unique and different knowledge into the project and that it is permitted to have different roles and contribute in different ways.*  *-It must not be a goal that everyone should contribute on an equal way and in the same extent*  *-User participation in research dissemination requires that one use time to talk about expectations, contributions and equivalence in the collaborations.*  *-I am afraid we are camouflaging the issue when everyone who uses the research is considered as users.*  *-I believe users should be the ones who have the diagnoses and who are ultimately the ones who should benefit from the research.*  *-We could invite the authorities for discussion. It`s a paradox that user-involvement is required, while research about the benefits of participation is lacking”* |
